# MicroRNA-770 affects proliferation and cell cycle transition by directly targeting CDK8 in glioma

**DOI:** 10.1186/s12935-018-0694-9

**Published:** 2018-12-03

**Authors:** Jun-feng Zhang, Jian-shui Zhang, Zhao-hua Zhao, Peng-bo Yang, Sheng-feng Ji, Nan Li, Qin-dong Shi, Jing Tan, Xi Xu, Cang-bao Xu, Ling-yu Zhao

**Affiliations:** 1 Shaanxi Key Laboratory of Ischemic Cardiovascular Disease, Institute of Basic and Translational Medicine, Xi’an Medical University, Xi’an, 710021 Shaanxi People’s Republic of China; 2 Department of Human Anatomy, Xi’an Medical University, Xi’an, 710021 Shaanxi People’s Republic of China; 3 Department of Human Anatomy, Histology and Embryology, Xi’an Jiaotong University Health Science Center, Xi’an, 710061 Shaanxi People’s Republic of China; 4 Department of Neuropathology, The First Affiliated Hospital of Xi’an Jiaotong University, Xi’an, 710061 Shaanxi People’s Republic of China; 5 The First Affiliated Hospital of Xi’an Jiaotong University, Xi’an, 710061 Shaanxi People’s Republic of China; 6 Department of Cell Biology and Genetics, School of Basic Medical Sciences, Xi’an Jiaotong University Health Science Center, Xi’an, 710061 People’s Republic of China

**Keywords:** miR-770, CDK8, Glioma, Proliferation, Cell cycle

## Abstract

**Background:**

MicroRNAs play crucial roles in tumorigenesis and tumor progression. miR-770 has been reported to be downregulated in several cancers and affects cancer cell proliferation, apoptosis, metastasis and drug resistance. However, the role and underlying molecular mechanism of miR-770 in human glioma remain unknown and need to be further elucidated.

**Methods:**

The expression of miR-770 in glioma tissues and cell lines was measured by quantitative real-time PCR (qRT-PCR) to explore the association of miR-770 expression with clinicopathological characteristics. The expression of CDK8 was detected by qRT-PCR and Western blotting in glioma tissues. A target prediction program and a dual-luciferase reporter assay were used to confirm that CDK8 is a target gene of miR-770. MTT and cell counting assays were used to assess the effect of miR-770 on glioma cell proliferation. The cell cycle distribution and apoptosis were examined by flow cytometry. CDK8 siRNA and overexpression were used to further confirm the function of the target gene.

**Results:**

We demonstrated that miR-770 expression was downregulated in human glioma tissues and cell lines. The overexpression of miR-770 inhibited glioma cell proliferation and cell cycle G1-S transition and induced apoptosis. The inhibition of miR-770 facilitated cell proliferation and G1-S transition and suppressed apoptosis. miR-770 expression was inversely correlated with CDK8 expression in glioma tissues. CDK8 was confirmed to be a direct target of miR-770 by using a luciferase reporter assay. The overexpression of miR-770 decreased CDK8 expression at both the mRNA and protein levels, and the suppression of miR-770 increased CDK8 expression. Importantly, CDK8 silencing recapitulated the cellular and molecular effects observed upon miR-770 overexpression, and CDK8 overexpression eliminated the effects of miR-770 overexpression on glioma cells. Moreover, both exogenous expression of miR-770 and silencing of CDK8 resulted in suppression of the Wnt/β-catenin signaling pathway.

**Conclusions:**

Our study demonstrates that miR-770 inhibits glioma cell proliferation and G1-S transition and induces apoptosis through suppression of the Wnt/β-catenin signaling pathway by targeting CDK8. These findings suggest that miR-770 plays a significant role in glioma progression and serves as a potential therapeutic target for glioma.

## Background

As the most common malignant primary tumors of the central nervous system, gliomas have high morbidity and mortality [1] and account for more than 70% of brain tumors [2, 3]. The incidence of glioma increased from 5.9 of 100, 000 people in 1973 to 6.61 of 100, 000 people in 2016, possibly as a consequence of improved radiological diagnosis [4]. Tremendous progress has been achieved in diagnosis and stratification of prognostication. Little progress has been achieved in treatment etc. Unfortunately, the five-year overall survival of glioma patients in advanced stages remains poor [5]. Therefore, it is critical to uncover the molecular mechanisms underlying glioma development and progression, and classification such as IDH status, ATRX/TERT, 1p/19q codeletion, histone gene mutations, which could reveal novel biomarkers and support the development of therapeutic strategies for patients with glioma.

MicroRNAs (miRNAs) are a family of single-stranded, endogenous, small noncoding RNA molecules of approximately 20 nucleotides [6] that act as key regulators of gene expression by binding to the 3′-untranslated regions (3′-UTR) of target mRNAs [7–9]. miRNAs can regulate gene expression by repressing translation or accelerating the degradation of mRNA [10]. By modulating different target genes, miRNAs play vital roles in diverse biological processes, such as tumor angiogenesis, cell proliferation, differentiation, stress responses, apoptosis, adhesion, glucose uptake, metastasis and resistance to cancer chemotherapy [11–15]. miRNAs can downregulate multiple target genes, including oncogenes and tumor suppressors; thus, some miRNAs function as tumor suppressors, and others function as oncogenes [16]. Accumulating evidence has shown that the dysregulation of miRNAs plays an important role in glioma progression. Recently, several studies have shown that miR-770 is clinically significant and plays a crucial role in carcinogenesis and cancer progression in a variety of cancers, such as breast cancer, non-small cell lung cancer, ovarian cancer and hepatocellular carcinoma [17–19]. Nevertheless, the role and molecular mechanism of miR-770 in human glioma development remain unknown and need to be further elucidated.

In this study, we found that the expression of miR-770 was significantly downregulated in glioma tissues and correlated with clinicopathological characteristics, such as miR-770 expression decreasing in IDH-mutated groups of glioma compared with IDH-wildtype. In addition, our results showed that cyclin-dependent kinase 8 (CDK8) was overexpressed in glioma tissues. We predicted that miR-770 could target CDK8 by using bioinformatics software (RegRNA). Furthermore, miR-770 potently inhibited human glioma cell proliferation and induced G1-S cell cycle arrest and cell apoptosis. More importantly, for the first time, we provided evidence that CDK8 is a direct functional target of miR-770. Our findings suggest that miR-770 may be a novel therapeutic target in glioma therapy.

## Methods

### Clinical samples and cell lines

Sixty-three glioma tissues and paired adjacent normal tissues were obtained from patients who were diagnosed in the Department of Neurosurgery, First Affiliated Hospital, Xi’an Jiaotong University, China. We obtained informed consent from each patient before specimen collection. The tissues were immediately frozen and stored at − 80 °C. Clinicopathological features were confirmed by a neuropathologist according to the WHO 2016 criteria. Isocitrate dehydrogenase (IDH)-1 mutation status was assessed by immunohistochemistry (IHC). When IHC results for IDH1 were negative, we tested for IDH1 mutations using the hot-spot technique. The codeletion of 1p/19q was evaluated by fluorescence in situ hybridization. The experiments were approved by the Ethics Committee of Xi’an Jiaotong University Health Science Center. Human glioma cell lines (IDH Mutation: SNB19; IDH wild type: LN229, U87, U251) and primary normal human astrocytes (NHAs) were purchased from the Cell Bank (Shanghai, China). These cells were maintained in Dulbecco’s modified Eagle’s medium (DMEM, Gibco, Grand Island, NY, USA) supplemented with 10% fetal bovine serum (Gibco), penicillin (100 U/mL), and streptomycin (100 μg/mL) and were incubated at 37 °C in a humidified atmosphere of 5% CO_2_ and 95% air.

### Isolation of total RNA and quantitative real-time PCR (qRT-PCR)

Total RNA was extracted from human glioma tissues and cell lines with TRIzol reagent (Thermo Fisher Scientific, Waltham, MA, USA). The SYBR Premix Ex Taq II Kit (Takara, China) was used to measure miR-770 expression and cyclin-dependent kinase (CDK) mRNA expression. qRT-PCR was performed by using the iCycler iQ Multicolor qRT-PCR System (Bio-Rad, USA). The data were normalized to RNU6B (U6) or β-actin gene expression. The primer sequences were as follows: miR-770 reverse-transcribed primer, 5′-GTCGTATCCAGTGCGTGTCGTGGAGTCGGCAATTGCACTGGATACGACAGGGCCA-3′; miR-770 forward, 5′-ATCCAGTGCGTGTCGTG-3′; miR-770 reverse, 5′-TGCTTCCAGTACCACGTGTC-3′; U6 reverse-transcribed primer, 5′-CGCTTCACGAATTTGCGTGTCAT-3′; U6 forward, 5′-GCTTCGGCAGCACATATACTAAAAT-3′; U6 reverse, 5′-CGCTTCACGAATTTGCGTGTCAT-3′; CDK8 forward, 5′-GCCGGTTGTCAAATCCCTTAC-3′; CDK8 reverse, 5′-TGTGACTGCTGTCTTGATTCCCT-3′; β-actin forward, 5′-TGGCACCCAGCACAATGAA-3′; and β-actin reverse, 5′-CTAAGTCATAGTCCGCCTAGAAGCA-3′.

### Expression vector construction

The Hsa-miR-770 precursor expression vector (named miR-770) and the control empty vector (named control) were constructed with synthetic oligonucleotides and incorporated into the pcDNA6.2-GW/EmGFPmiR plasmid according to the manufacturer’s instructions. Full-length human CDK8 complementary DNA was cloned into the pCMV2-GV146 vector. Transfection was performed using Lipofectamine 2000 (Invitrogen, Carlsbad, CA, USA) according to the manufacturer’s instructions.

### Dual-luciferase assay

The binding site for miR-770 in the 3′-UTR of CDK8 was constructed with synthetic oligonucleotides (Beijing AuGCT DNA-SYN Biotechnology, China) and cloned into the pmirGLO Dual-Luciferase expression vector (named CDK8-WT). The mutated 3′-UTR sequences of CDK8 were also cloned and named CDK8-MT. The pre-miR-770 expression vector and the WT or MT reporter plasmids were cotransfected into HEK293T cells. The cells were harvested 24 h after transfection. The Dual-Luciferase Assay System (Promega, Madison, USA) was used to detect reporter activity according to the manufacturer’s protocol.

### Anti-miR-770/CDK8 siRNA synthesis and transfection

Interfering RNA oligonucleotides served as miR-770 inhibitors (named anti-miR-770) and were synthesized by Gene Pharma (Shanghai, China). The sequence of anti-miR-770 was 5′-UGGCCCUGACACGUGGUACUGGA-3′. Scramble siRNA was used as a control (named anti-miR-Control), and the sequence was 5′-CAGUACUUUUGUGUAGUACAA-3′. The inhibitors were transfected into human glioma U251 cells with Lipofectamine 2000. Small interfering RNA (siRNA) was used to silence the human CDK8 gene. CDK8 siRNA (CDK8: sc-29267, Santa Cruz) and negative control siRNA (NC-siRNA: sc-37007, Santa Cruz) were transfected using Lipofectamine 2000 and diluted to a concentration of 50 nM for use in future experiments in U251 cells.

### MTT assay

Human glioma U251 cells (5000 cells/well in 200 μL of DMEM medium) were seeded into three 96-well plates (5-parallel wells/group) and cultured for 24 h. Then, the cells were treated with control, miR-770, anti-miR-Control, anti-miR-770, NC-siRNA (50 nM), CDK8 siRNA (50 nM), vector control and the CDK8 overexpression vector for 24, 48 and 72 h, respectively. Cell viability was detected with the MTT assay on a Versamax microplate reader (Molecular Devices, Sunnyvale, CA, USA) at a wavelength of 492 nm.

### Cell counting assay

To measure cell proliferation, 2.5 × 10^5^ cells were plated in 60-mm-diameter plates and cultured for 24 h. U251 cells were treated separately with control, miR-770, anti-miR-Control, anti-miR-770, NC-siRNA (50 nM), CDK8 siRNA (50 nM), vector control and the CDK8 overexpression vector. The number of cells was calculated at 24, 48 and 72 h after treatment with a Countess automated cell counter (Life Technologies Corp., Carlsbad, USA).

### Cell cycle analysis

The U251 cells were cultured in 6-well plates and treated for 48 h. Then, the cells were harvested and fixed in 70% ice-cold ethanol at 4 °C. The fixed cells were washed in PBS and stained with 50 μg/mL propidium iodide containing 50 μg/mL RNase A (DNase-free) for 15 min at room temperature. Next, the cells were subjected to fluorescence-activated cell sorting (BD Biosciences, USA). Different cell cycle populations were analyzed by using ModFit software.

### Apoptosis assay

U251 cells were seeded into 6-well plates and treated for 48 h. We examined cell apoptosis with an Annexin-V FITC Apoptosis Detection Kit (Invitrogen, USA) according to the manufacturer’s instructions. Apoptotic cells were measured by using a flow cytometer (BD Biosciences, USA). ModFit software was used to analyze apoptotic changes.

### Western blotting

We performed Western blotting according to standard methods. Briefly, tissue samples and glioma cells were lysed using lysis buffer (Wolsen, China) and centrifuged at 12,000*g* at 4 °C. The protein concentration was examined with the bicinchoninic acid (BCA) assay. The total protein was separated via 10% SDS-PAGE and electrophoretically transferred onto PVDF membranes (Invitrogen, Carlsbad, CA, USA). The membranes were incubated for 1 h in blocking solution containing 5% nonfat dry milk and then incubated with primary antibodies overnight at 4 °C. The primary antibodies were as follows: mouse polyclonal anti-CDK8 (1:1000, Cell Signaling Technology, USA), rabbit monoclonal anti-β-catenin (1:1000, Santa Cruz, CA, USA), mouse monoclonal anti-cyclin D1 (1:1000, Santa Cruz, CA, USA), and mouse monoclonal anti-β-actin (1:5000, Santa Cruz, CA, USA). The blots were developed with an ECL chemiluminescence kit (Pierce, Rockford, IL, USA). The blots were scanned, and the band densities were analyzed using PDQuest software.

### Statistical analysis

All experiments were performed at least 3 times independently. All data were analyzed using SPSS 20.0 software (Abbott Laboratories, Chicago, IL). The statistical significance of differences between groups was analyzed with one-way ANOVA or Student’s t-test. A Chi square test was employed to analyze the relationships between miR-770 expression and clinicopathologic characteristics. Correlation analysis between miR-770 and CDK8 in glioma tissues was performed using Pearson’s correlation analysis. The data are presented as the mean ± standard error mean (SEM) from 3 independent experiments. Values of p < 0.05 were considered to indicate statistically significant differences.

## Results

### miR-770 is significantly downregulated in human glioma tissues and cell lines

**Fig. 1:**
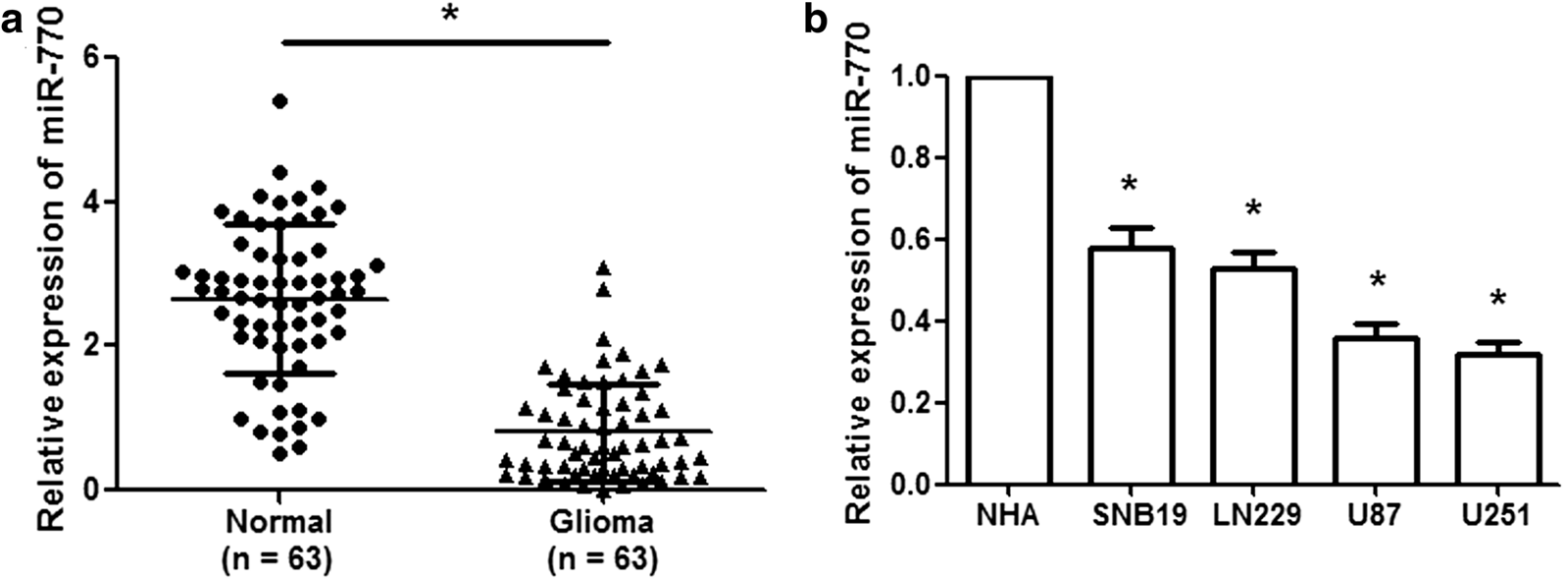
miR-770 is downregulated in glioma tissues and cell lines. **a** miR-770 expression was remarkably decreased in glioma tissues compared with that in adjacent normal tissues. **b** MiR-770 expression was significantly reduced in glioma cell lines (SNB19, LN229, U87 and U251) compared with that in normal human astrocyte (NHA) cells. *p < 0.01

To analyze the expression status of miR-770 in human glioma tissues, we performed qRT-PCR to examine miR-770 expression in clinical samples (63 glioma tissues and adjacent normal tissues) and glioma cell lines. The qRT-PCR assays showed that miR-770 expression was remarkably lower in glioma tissues than in adjacent normal tissues (Fig. 1a; p < 0.01). Subsequently, we investigated the correlations between miR-770 expression and the clinicopathological characteristics of glioma patients. As shown in Table 1, low miR-770 expression was associated with an advanced WHO pathological grade of glioma (p < 0.001), IDH1 mutation (p < 0.001) and a high KPS score (p < 0.001). However, miR-770 expression was not associated with gender, age, 1p/19q codeletion or tumor size. Furthermore, miR-770 expression was significantly lower in glioma cell lines (SNB19, LN229, U87 and U251) than in NHA cells (Fig. 1b; p < 0.01). These results indicated that miR-770 might be an effective biomarker for the diagnosis and detection of glioma.

**Table 1:** The correlation between miR-770 expression and clinicopathological characteristics in glioma patients

Characteristics	All patients	miR-770 expression		*P* value
		High (n = 11)	Low (n = 52)	
Gender				0.697
Male	39	7	32	
Female	24	4	20	
Age (years)				0.735
≥ 50	35	6	29	
< 50	28	5	23	
WHO grade				< 0.001
I + II	23	8	15	
III + IV	40	3	37	
IDH1				< 0.001
Mutation	29 (Nod:13; Cod:16)	2	27	
Wild type	34 (Nod:28; Cod:6)	9	25	
1p/19q				0.053
No deletion	41	6	35	
Codeletion	22	5	17	
Tumor size (cm)				0.163
≥ 5	36	5	31	
< 5	27	6	21	
KPS score				< 0.001
< 80	29	8	21	
≥ 80	34	3	31	

*Nod* no deletion, *Cod* codeletion

### miR-770 inhibits U251 glioma cell proliferation, prohibits cell cycle transition and exacerbates apoptosis

**Fig. 2:**
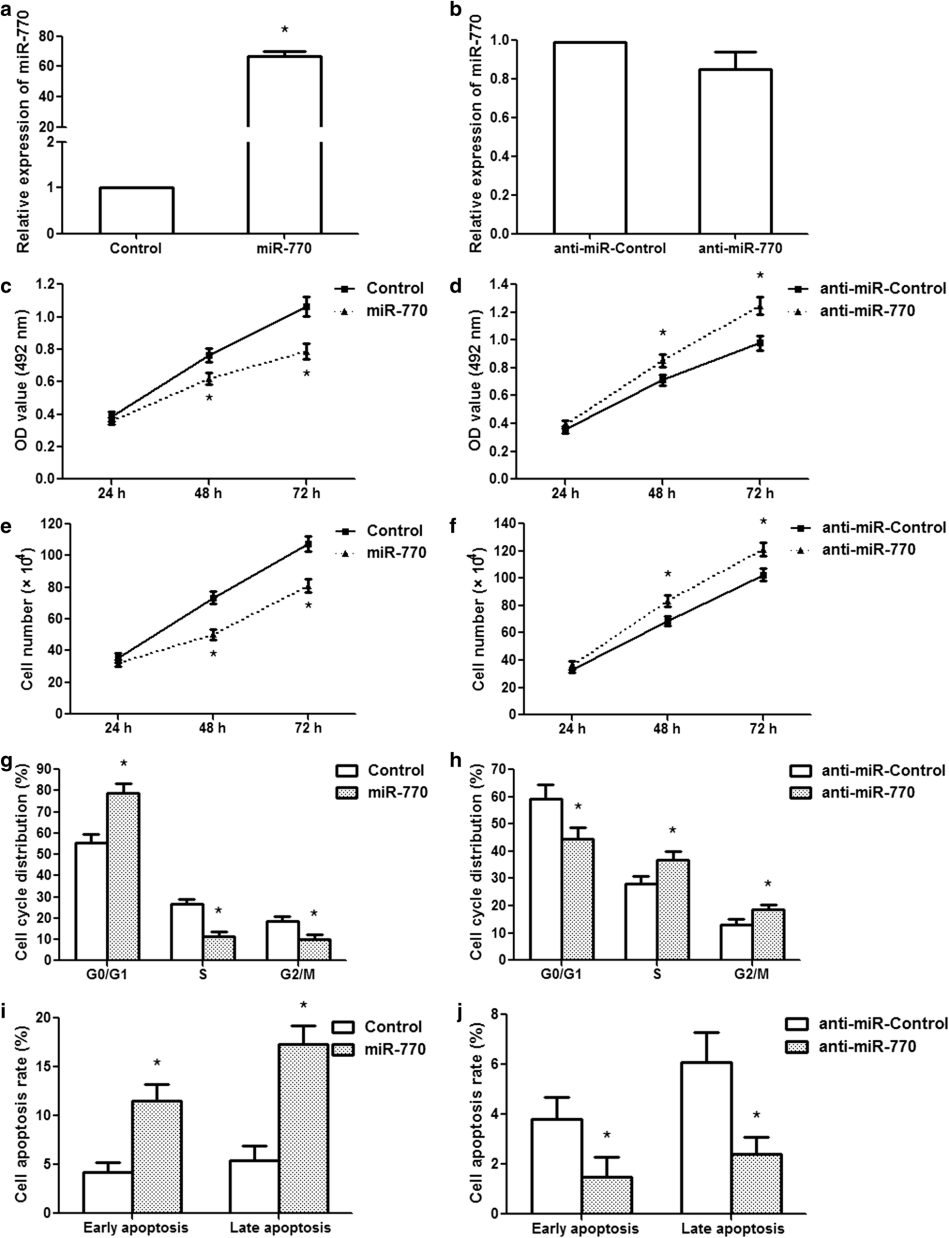
miR-770 inhibits human glioma U251 cell proliferation and induces G1-S cell cycle arrest and apoptosis. **a** miR-770 expression was detected in U251 cells after miR-770 overexpression. **b** miR-770 expression was examined in U251 cells after anti-miR-770 treatment. **c** miR-770 overexpression decreased cell activity at 48 and 72 h after transfection. **d** Anti-miR-770 increased cell activity at 48 and 72 h after transfection. **e** miR-770 overexpression suppressed glioma cell proliferation. **f** Anti-miR-770 promoted glioma cell growth. **g** The histogram represents the proportion of cells in the G0/G1, S and G2/M phases after miR-770 overexpression. **h** The ratio of cells in the G0/G1, S and G2/M phases after anti-miR-770 transfection. **i** The data revealed the ratios of early and late apoptosis after miR-770 overexpression. **j** The data showed the proportions of early apoptosis and late apoptosis after anti-miR-770 transfection. *p < 0.01, n = 3

To investigate the role of miR-770 in human glioma, U251 cells were transfected with the miR-770 precursor expression vector, a control empty vector, miR-770 antisense oligonucleotides, or the negative control. miR-770 expression was detected by qRT-PCR after transfection. miR-770 expression was significantly increased in cells transfected with the miR-770 vector compared to that in cells transfected with the control vector (p < 0.01); however, there were no prominent differences between the anti-miR-770 group and the anti-miR-Control group (Fig. 2a, b). An MTT assay revealed that miR-770 overexpression remarkably suppressed the proliferation of U251 cells at 48 and 72 h after transfection (Fig. 2c; p < 0.01), while anti-miR-770 promoted cell growth at 48 and 72 h after transfection (Fig. 2d; p < 0.01). A similar trend was observed in the cell counting assay. miR-770 overexpression inhibited cell proliferation, but anti-miR-770 promoted cell growth (Fig. 2e, f; p < 0.01). Because cell cycle is involved in the regulation of cell proliferation, we measured this processe using a flow cytometer. The results showed that miR-770 overexpression resulted in a remarkable accumulation of the G0/G1 phase population and a reduction of the S and G2/M phase populations in U251 cells (Fig. 2g; p < 0.01); reduction of miR-770 significantly decreased the G0/G1 phase population and increased the S and G2/M phase populations (Fig. 2h; p < 0.01). Evaluation of cell apoptosis confirmed that the ratio of early-apoptotic to late-apoptotic cells was clearly increased when miR-770 was overexpressed (Fig. 2i; p < 0.01) and remarkably decreased when anti-miR-770 was transfected (Fig. 2j; p < 0.01). These results demonstrated that miR-770 reduced glioma cell proliferation and induced G1-S cell cycle arrest and apoptosis.

### CDK8 is a target gene of miR-770

**Fig. 3:**
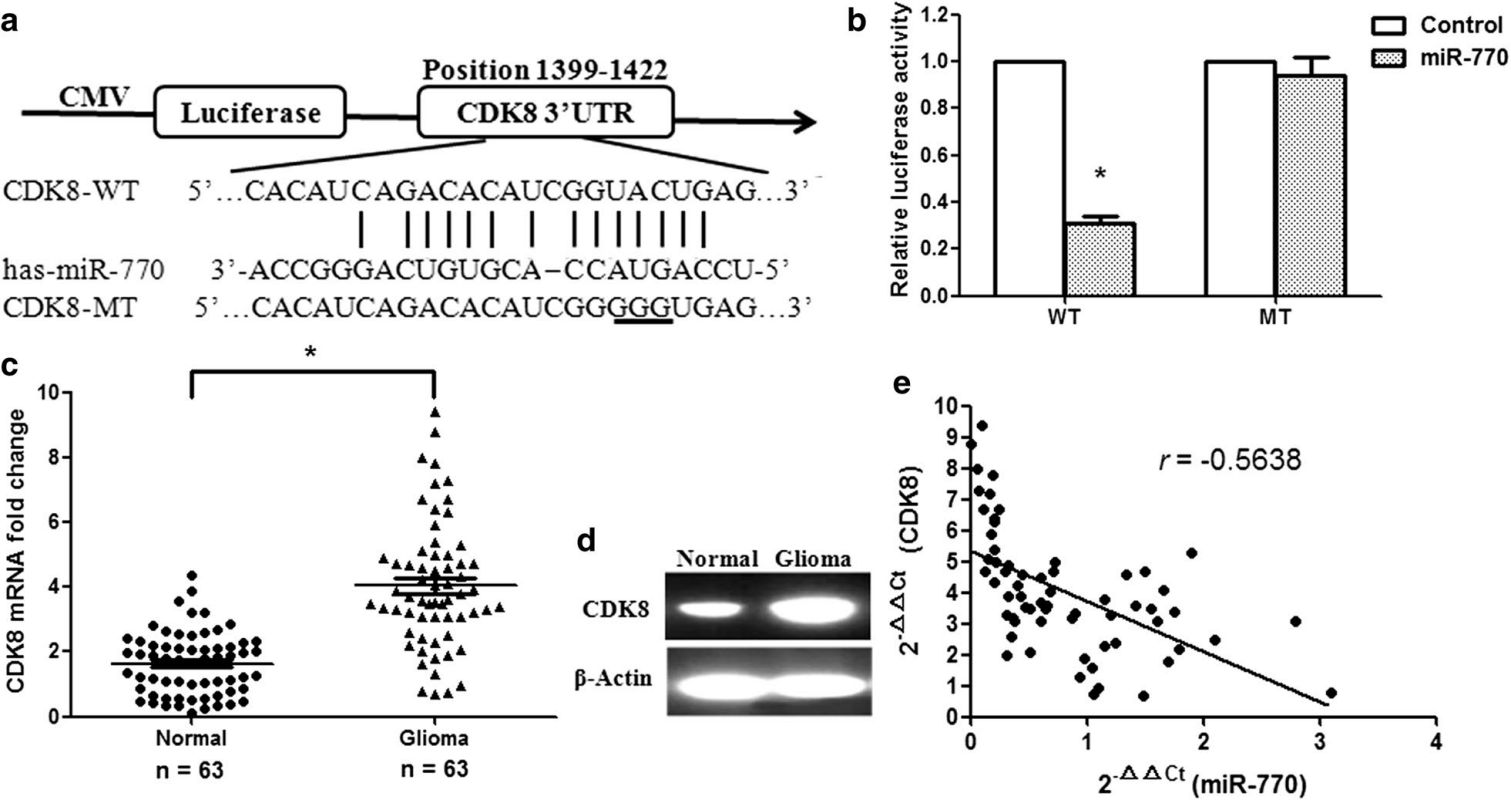
miR-770 directly targets the CDK8 gene. **a** Bioinformatics predicted interactions of miR-770 and its binding sites in the 3′-UTR of CDK8. **b** Luciferase activity was examined by the dual-luciferase assay. **c** CDK8 mRNA expression in human glioma tissues. **d** CDK8 protein levels were measured by Western blotting. **e** miR-770 and CDK8 levels were inversely correlated. The 2^−ΔΔCt^ values of miR-770 and CDK8 were subjected to a Pearson correlation analysis (n = 63, r = − 0.5638, p < 0.001, Pearson’s correlation). *p < 0.01

A bioinformatic database (RegRNA) was used to confirm a large number of possible target genes of miR-770. CDK8 was selected from these candidates for further study. We found that there was a binding site for miR-770 in the 3′-UTR of the CDK8 mRNA ranging from 1399 to 1422 bp (Fig. 3a). To determine whether miR-770 directly targets CDK8, a dual-luciferase reporter system containing the WT and MT 3′-UTR of CDK8 was used. HEK293T cells were cotransfected with reporter plasmids and pre-miR-770 or the pmirGLO empty vector (control). Pre-miR-770/WT-CDK8-UTR-transfected cells showed a remarkable reduction in luciferase activity (p < 0.01), and pre-miR-770/MT-CDK8-UTR-transfected cells failed to exhibit reduced relative luciferase activity (Fig. 3b), suggesting that miR-770 directly targets the 3′-UTR of CDK8. Next, we measured CDK8 expression at the mRNA and protein levels. Our results showed that the expression of CDK8 was significantly upregulated at both the mRNA and protein levels in glioma tissues compared to that in adjacent normal tissues (Fig. 3c, d; p< 0.01). The effect of miR-770 on CDK8 was assessed based on the data obtained from qRT-PCR. A significant negative correlation was identified between CDK8 and miR-770 (Fig. 3e; n = 63, r = − 0.5638, p < 0.001, Pearson’s correlation).

### miR-770 suppresses glioma cell growth and induces G1-S arrest through the β-catenin signaling pathway by targeting CDK8

**Fig. 4:**
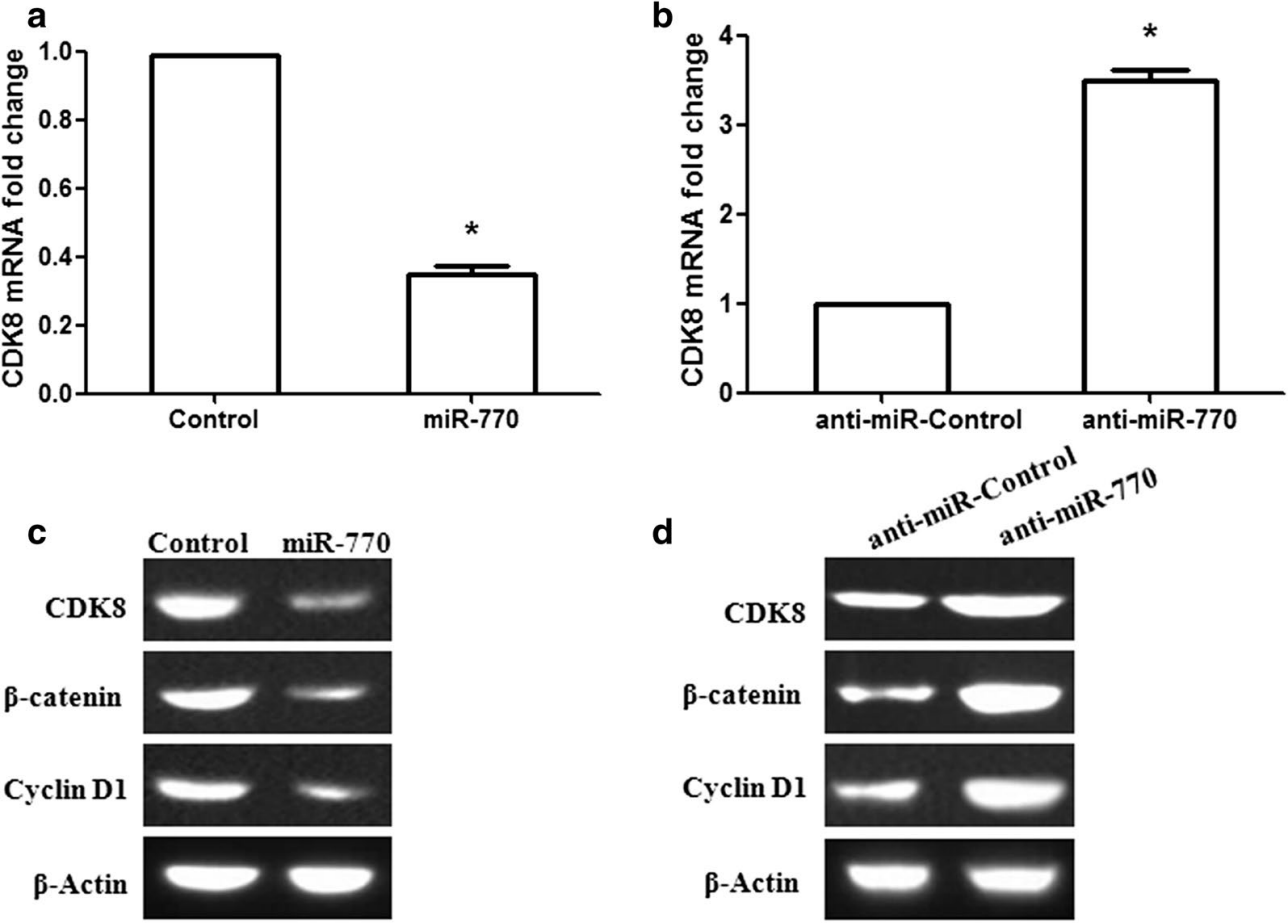
miR-770 regulates the Wnt/β-catenin signaling pathway in human glioma cells by targeting CDK8. **a** CDK8 mRNA was determined in U251 cells after miR-770 overexpression. **b** CDK8 mRNA was examined in U251 cells after anti-miR-770 treatment. **c** miR-770 overexpression inhibited the expression of the CDK8, β-catenin and cyclin D1 proteins in U251 cells. **d** Anti-miR-770 promoted CDK8, β-catenin and cyclin D1 protein expression. *p < 0.001

miR-770 overexpression significantly decreased the mRNA expression of CDK8 in U251 cells, while anti-miR-770 remarkably increased CDK8 mRNA expression (Fig. 4a, b; p< 0.001). A similar trend was observed for protein levels (Fig. 4c, d). To further investigate the latent molecular mechanisms of miR-770-regulated cell proliferation and cell cycle transition, we examined the protein levels of the related Wnt/β-catenin signaling pathway and the G1 regulator cyclin D1 by using Western blot analysis. Our results showed that miR-770 overexpression downregulated β-catenin and cyclin D1 protein expression levels in U251 cells (Fig. 4c). In contrast, anti-miR-770 upregulated β-catenin and cyclin D1 protein expression (Fig. 4d). These results demonstrated that miR-770 could modulate glioma cell proliferation and the cell cycle through regulating the Wnt/β-catenin signaling pathway.

### Silencing of CDK8 restrains glioma cell proliferation

**Fig. 5:**
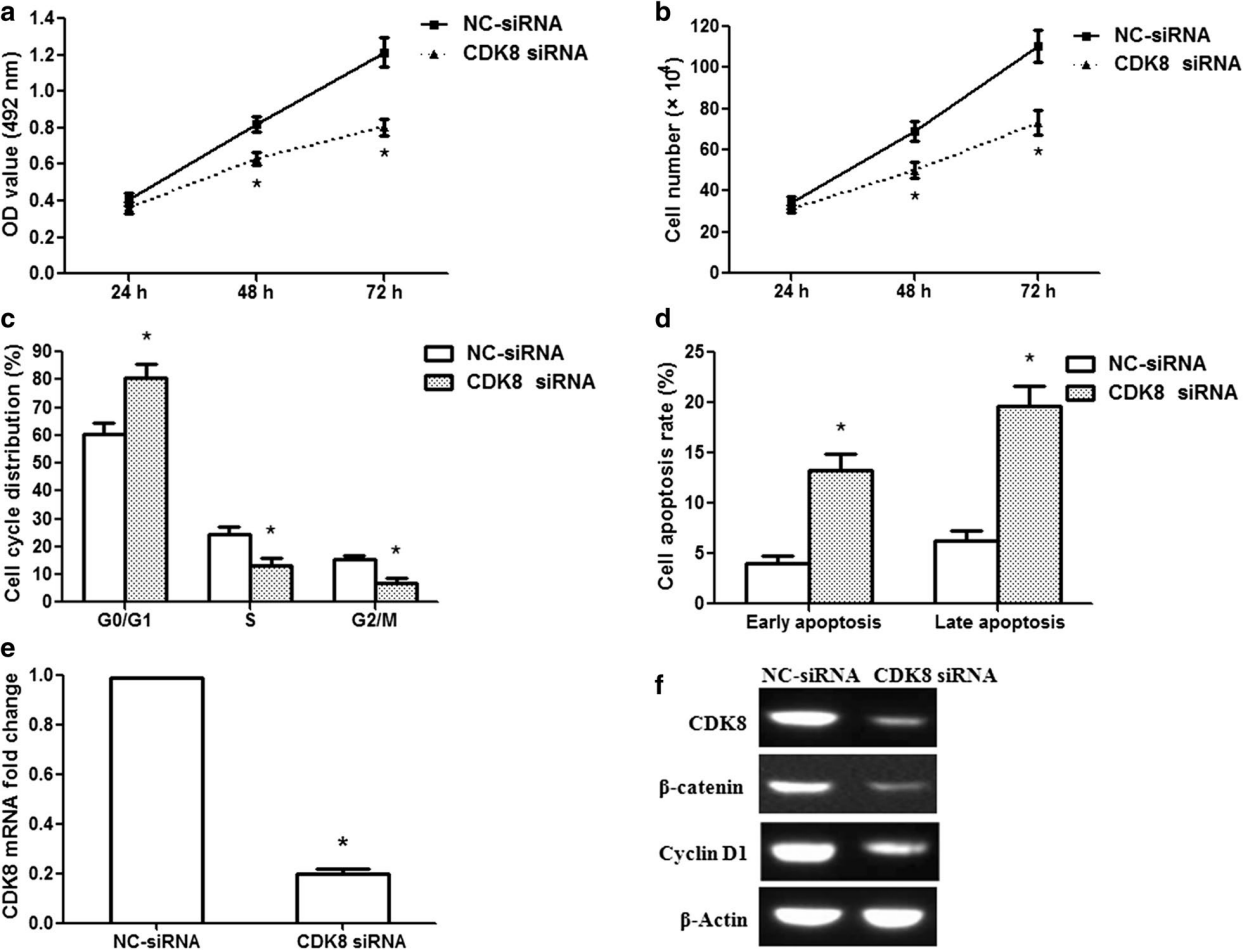
CDK8 siRNA suppresses the proliferation of human glioma U251 cells. **a** CDK8 siRNA decreased the activity of U251 cells at 48 and 72 h. **b** CDK8 siRNA inhibited U251 cell proliferation. **c** Flow cytometric analysis showed the percentage of U251 cells in the G0/G1, S and G2/M phases. G0/G1 phase cells increased after CDK8 siRNA treatment, and S and G2/M phase cells were reduced. **d** The data showed the percentage of early and late apoptosis after CDK8 siRNA treatment. **e** The knockdown efficiency of CDK8 siRNA in U251 cells. **f** CDK8, β-catenin and cyclin D1 protein expression was examined after CDK8 siRNA treatment. *p < 0.01, n = 3

Since miR-770 regulated cell proliferation, the cell cycle and apoptosis in glioma cells, CDK8 was validated as a direct target of miR-770, therefore, CDK8 was knocked down in glioma cells by RNA interference to validate its involvement in the tumor suppressor functions of miR-770. Silencing of CDK8 significantly decreased cell activity at 48 and 72 h after transfection (Fig. 5a; p< 0.01). A cell counting assay also revealed that silencing of CDK8 remarkably inhibited U251 cell proliferation (Fig. 5b; p< 0.01). Silencing of CDK8 increased the G0/G1 phase population and reduced the S and G2/M phase populations in U251 cells (Fig. 5c; p< 0.01). Furthermore, silencing of CDK8 induced apoptosis in U251 cells (Fig. 5d; p< 0.01). Next, we analyzed the knockdown efficiency of CDK8 siRNA at the mRNA and protein levels. Our results showed that CDK8 mRNA expression was specifically knocked down in U251 cells by the siRNA (Fig. 5e; p< 0.01). The protein expression of CDK8 decreased significantly in the siRNA group compared with that in the NC-siRNA group, and β-catenin and cyclin D1 protein expression levels were also reduced (Fig. 5f). These findings were similar to those obtained after miR-770 overexpression, indicating a similar effect of CDK8 knockdown and miR-770 overexpression.

### Overexpression of CDK8 eliminated the effects of miR-770 on U251 glioma cells

**Fig. 6:**
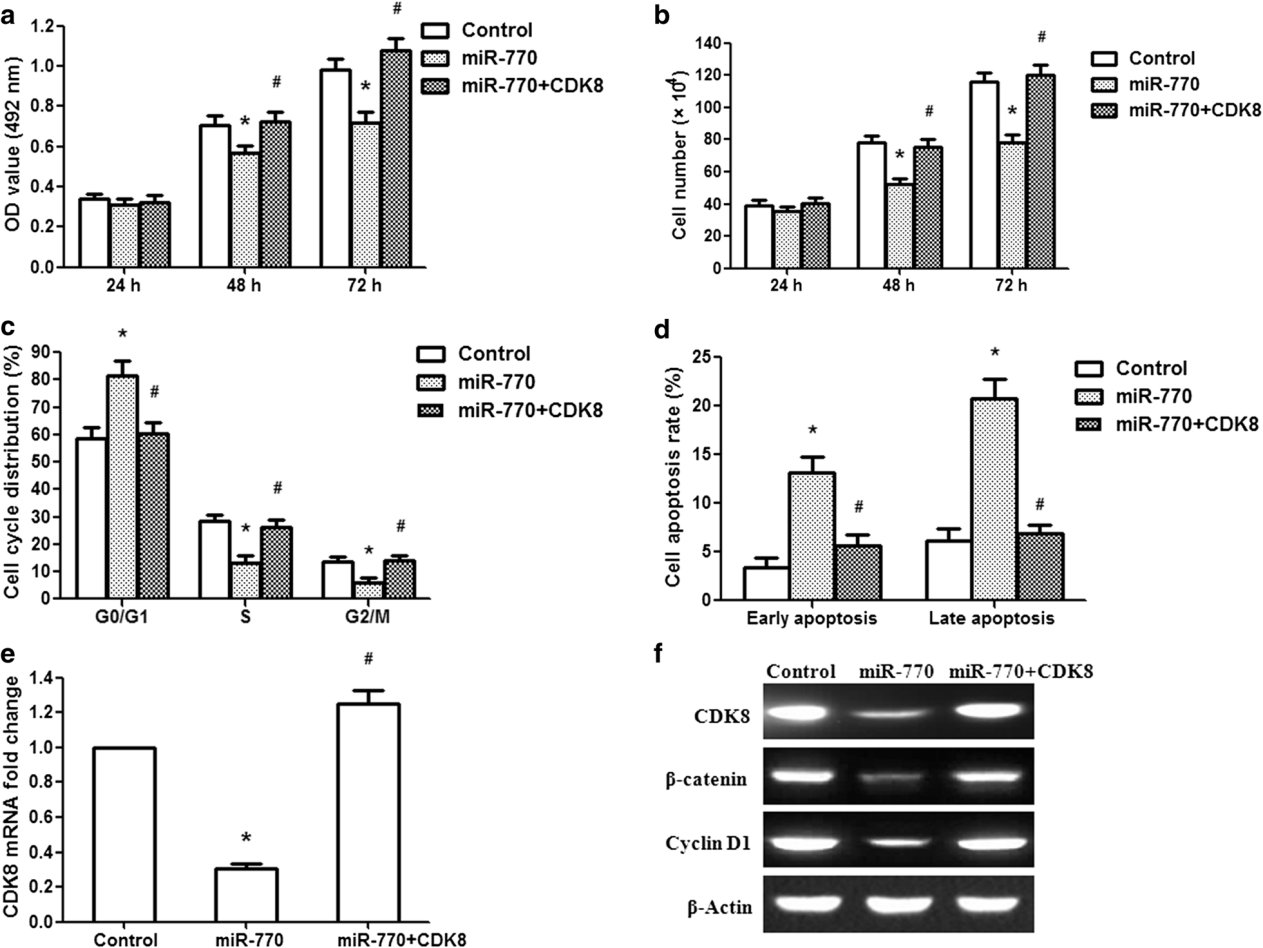
CDK8 overexpression rescues miR-770-induced cellular phenotypes in glioma cells. **a** U251 cell activity was measured after cotransfection with CDK8 and miR-770 vectors. **b** U251 cell proliferation was examined after cotransfection with CDK8 and miR-770 vectors. **c** Cell cycle was determined in U251 cells at 48 h. **d** Apoptosis was detected in U251 cells at 48 h. **e** CDK8 overexpression rescued CDK8 mRNA expression levels reduced by miR-770. **f** CDK8, β-catenin and cyclin D1 protein expression was examined after cotransfection with CDK8 and miR-770 vectors. *p < 0.01, compared with the vector control group; #p < 0.01, compared with the miR-770 overexpression group. n = 3

To further confirm that miR-770 performed a tumor suppressor function via CDK8, we constructed a CDK8 overexpression vector, which was cotransfected with miR-770 into U251 cells. After cotransfection with the miR-770 and CDK8 vectors, we found that the overexpression of CDK8 counterbalanced the tumor suppressor effect of miR-770 in glioma cells during cell proliferation (Fig. 6a, b). The effect of CDK8 overexpression on cell cycle progression was examined by flow cytometry. The results showed that overexpression of CDK8 induced U251 cells to re-enter the S and G2/M phases (Fig. 6c). Furthermore, CDK8 overexpression eliminated the impact of miR-770 on U251 cell apoptosis (Fig. 6d). Overexpression of CDK8 in U251 cells rescued the reduced CDK8 mRNA expression levels induced by miR-770 (Fig. 6e). Further analysis revealed that compared with miR-770 overexpression, the overexpression of CDK8 upregulated CDK8, β-catenin and cyclin D1 protein expression (Fig. 6f). These findings further demonstrated that miR-770 plays a tumor suppressor role through the Wnt/β-catenin signaling pathway by targeting CDK8.

## Discussion

In recent decades, emerging evidence has demonstrated that miRNAs are actively involved in the pathogenesis of cancers [20]. Many miRNAs have been identified by microarray screening in gliomas [21–23]. In addition, miRNAs have been found to be key regulators of glioma cell proliferation, differentiation, apoptosis, metastasis, invasion and epithelial-mesenchymal transition [24–27]. Due to the crucial roles of miRNAs in glioma, miRNAs have been proposed as prospective biomarkers and therapeutic targets of glioma [28]. Although the clinical significance of miRNAs has been well characterized in glioma, the roles and the underlying molecular mechanisms of dysregulated miRNAs remain unknown. Therefore, identifying miRNAs and elucidating their biological functions in glioma will help identify novel targets for diagnosis and therapy. Recent papers reported the downregulation of miR-770 in non-small cell lung cancer and gastric cardia adenocarcinoma [18, 29]. It has been reported that miR-770 can suppress the chemo-resistance and metastasis of triple-negative breast cancer via direct targeting of STMN1 [17]. miR-770 functions as an anti-oncogene and promotes chemosensitivity in ovarian cancer by downregulating ERCC2 [30]. Moreover, miR-770 inhibited tumorigenesis and EMT by targeting JMJD6 and regulating the WNT/β-catenin pathway in non-small cell lung cancer [18]. However, the exact functions and mechanisms of miR-770 during tumorigenesis in human glioma remain unclear. In the present study, we found that miR-770 expression was dramatically downregulated in both glioma tissues and cell lines. The clinicopathological significance of miR-770 expression was also analyzed. The results revealed that low miR-770 levels were significantly associated with WHO pathological grade, IDH1 status and KPS score in glioma patients. Interestingly, miR-770 expression decreased in IDH-mutated groups of glioma compared with IDH-wildtype, suggesting that miR-770 may play an importance role in glioma diagnosis. The experiment demonstrated that miR-770 remarkably inhibited glioma cell growth by inducing G1-S phase arrest and promoting cell apoptosis. Our findings suggest that miR-770 plays a key role in glioma development and progression.

Furthermore, our miR-770 target analysis identified CDK8 as a direct target of miR-770. CDKs play crucial roles in regulating cell proliferation and differentiation in eukaryotes [31, 32]. The dysregulation of CDKs and their regulatory partners (cyclins) disrupts cell proliferation, differentiation and apoptosis, resulting in abnormal development, tumorigenesis, etc. There are 21 CDK family members in mammals [33]. Previous studies have shown the roles of several CDKs, such as CDK1, CDK2, CDK4 and CDK6, in regulating cell proliferation and contributing to tumorigenesis [34]. CDK8 is a serine-threonine protein kinase that is localized to the nucleus and controls gene expression by interacting with the transcriptional machinery and regulating RNAPII activity [35]. In this study, we found that CDK8 was overexpressed in glioma compared with the level in normal tissues, which revealed an inverse correlation between CDK8 mRNA expression and miR-770 expression in glioma tissues. These findings implied that miR-770 might affect the progression of glioma by targeting CDK8. Further bioinformatic analysis showed that there was an miR-770-binding site at 1399–1422 nt of the CDK8 3′-UTR. The dual-luciferase reporter assay demonstrated that miR-770 directly targeted CDK8 by recognizing the 3′-UTR of CDK8 mRNA and inhibited CDK8 translation. CDK8 has been shown to be involved in tumorigenesis and development. Evidence has demonstrated that CDK8 plays an oncogenic role in cancers, by stimulating epithelial-to-mesenchymal transition in pancreatic cancer [36], facilitating migration and invasion in prostate cancer [37], and promoting proliferation and metastasis in breast cancer [38], melanoma and colorectal cancers [33, 39]. The results also demonstrated that knockdown of CDK8 suppressed glioma cell proliferation by inducing G1-S phase arrest and cell apoptosis. Moreover, we found that the overexpression of CDK8 counterbalanced the tumor suppressor effect of miR-770 in glioma cells. These results further verify that miR-770 functions as a tumor suppressor in glioma by inhibiting CDK8 expression.

CDK8 is essential for β-catenin-dependent oncogenesis and progression in all kinds of cancers [39–41]. On the one hand, CDK8 can directly regulate β-catenin-activated transcription. For example, the β-catenin transcriptional program can be stimulated by a mediator complex, which includes CDK8, MED12, MED13 and its cyclin cofactor cyclin C [42, 43]. On the other hand, it has been demonstrated that CDK8 can directly phosphorylate E2F1 at S375 and then block the inhibitory effect of E2F1 on β-catenin transcription [44, 45]. The Wnt/β-catenin pathway is a conserved signaling pathway that is crucial for initiating and regulating a diverse range of biological processes, including embryogenesis, carcinogenesis, cell growth, apoptosis, and cell polarity [46–48]. For example, activation of the Wnt/β-catenin signaling pathway may facilitate the proliferation of embryonic, intestinal, skin, and neural stem cells, inducing the self-renewal and differentiation of stem-like cells [49–51]. The Wnt/β-catenin downstream regulator cyclin D1 is a crucial transcriptional factor in the G0/G1 phase [52]. Cyclin D1-CDK4/6 protein kinase complexes can regulate the cellular progression from G0/G1 phase to S phase [53]. It was reported that cyclin D1 is involved in human tumorigenesis. The results demonstrated that miR-770 overexpression and CDK8 siRNA could inhibit the expression of cyclin D1 and induce G1-S phase arrest by suppressing the Wnt/β-catenin signaling pathway. In contrast, anti-miR-770 increased the expression of cyclin D1 and drove more cells into the S and G2/M phases through activating the Wnt/β-catenin signaling pathway. Furthermore, CDK8 overexpression eliminated the effects of miR-770 overexpression on glioma cells. Our findings suggest that miR-770 inhibits G1-S phase transition through inhibition of the Wnt/β-catenin signaling pathway by targeting CDK8.

The growth rate of cancer tissues is determined by cell proliferation and cell apoptosis. An imbalance of apoptosis-related proteins may induce the dysregulation of apoptosis, which leads to oncogenesis and tumor development. Previous studies found that CDK8 prevented apoptosis in pancreatic, melanoma and colorectal cancers [33, 36]. We provide evidence that miR-770 induces glioma cell apoptosis through targeting CDK8.

## Conclusions

In summary, our study demonstrates that miR-770 functions as a tumor suppressor gene in glioma. We found that miR-770 is downregulated and associated with the clinicopathologic characteristics of glioma patients. miR-770 inhibits glioma cell proliferation and induces apoptosis through suppression of the Wnt/β-catenin signaling pathway by targeting CDK8. These findings suggest that miR-770 plays a significant role in glioma progression and may serve as a potential novel target for glioma therapy.

## Abbreviations

miRNAs: microRNAs
3′-UTR: 3′-untranslated region
miR-770: microRNA-770
CDK8: cyclin-dependent kinase 8
NHAs: normal human astrocytes
DMEM: Dulbecco’s modified Eagle’s medium
qRT-PCR: quantitative real-time PCR
siRNA: small interfering RNA

## References

[1] Reardon DA, Rich JN, Friedman HS, Bigner DD (2006). Recent advances in the treatment of malignant astrocytoma. J Clin Oncol.

[2] Siegel RL, Miller KD, Jemal A (2015). Cancer statistics, 2015. CA Cancer J Clin.

[3] Liang A, Zhou B, Sun W (2017). Integrated genomic characterization of cancer genes in glioma. Cancer Cell Int.

[4] McNeill KA (2016). Epidemiology of brain tumors. Neurol Clin.

[5] Li YN, Cao YQ, Wu X, Han GS, Wang LX, Zhang YH, Chen X, Hao B, Yue ZJ, Liu JM (2015). The association between Salt-inducible kinase 2 (SIK2) and gamma isoform of the regulatory subunit B55 of PP2A (B55gamma) contributes to the survival of glioma cells under glucose depletion through inhibiting the phosphorylation of S6K. Cancer Cell Int.

[6] Liu L, Cui S, Zhang R, Shi Y, Luo L (2017). MiR-421 inhibits the malignant phenotype in glioma by directly targeting MEF2D. Am J Cancer Res..

[7] Zhao LY, Tong DD, Xue M, Ma HL, Liu SY, Yang J, Liu YX, Guo B, Ni L, Liu LY (2017). MeCP2, a target of miR-638, facilitates gastric cancer cell proliferation through activation of the MEK1/2-ERK1/2 signaling pathway by upregulating GIT1. Oncogenesis..

[8] Hyrina A, Olmstead AD, Steven P, Krajden M, Tam E, Jean F (2017). Treatment-induced viral cure of hepatitis c virus-infected patients involves a dynamic interplay among three important molecular players in lipid homeostasis: circulating microRNA (miR)-24, miR-223, and proprotein convertase subtilisin/kexin type 9. EBioMedicine..

[9] Zhao LY, Yao Y, Han J, Yang J, Wang XF, Tong DD, Song TS, Huang C, Shao Y (2014). miR-638 suppresses cell proliferation in gastric cancer by targeting Sp2. Dig Dis Sci.

[10] Bushati N, Cohen SM (2007). microRNA functions. Annu Rev Cell Dev Biol.

[11] Kotaja N (2014). MicroRNAs and spermatogenesis. Fertil Steril.

[12] Imbar T, Eisenberg I (2014). Regulatory role of microRNAs in ovarian function. Fertil Steril.

[13] Jiang R, Zhang C, Liu G, Gu R, Wu H (2017). MicroRNA-101 inhibits proliferation, migration and invasion in osteosarcoma cells by targeting ROCK1. Am J Cancer Res..

[14] Garg D, Cohen SM (2014). miRNAs and aging: a genetic perspective. Ageing Res Rev..

[15] Dogini DB, Pascoal VD, Avansini SH, Vieira AS, Pereira TC, Lopes-Cendes I (2014). The new world of RNAs. Genet Mol Biol..

[16] Ruvkun G (2006). Clarifications on miRNA and cancer. Science.

[17] Li Y, Liang Y, Sang Y, Song X, Zhang H, Liu Y, Jiang L, Yang Q (2018). MiR-770 suppresses the chemo-resistance and metastasis of triple negative breast cancer via direct targeting of STMN1. Cell Death Dis..

[18] Zhang Z, Yang Y, Zhang X (2017). MiR-770 inhibits tumorigenesis and EMT by targeting JMJD6 and regulating WNT/β-catenin pathway in non-small cell lung cancer. Life Sci.

[19] Wu WJ, Shi J, Hu G, Yu X, Lu H, Yang ML, Liu B, Wu ZX (2016). Wnt/β-catenin signaling inhibits FBXW7 expression by upregulation of microRNA-770 in hepatocellular carcinoma. Tumour Biol.

[20] Tung SL, Huang WC, Hsu FC, Yang ZP, Jang TH, Chang JW, Chuang CM, Lai CR, Wang LH (2017). miRNA-34c-5p inhibits amphiregulin-induced ovarian cancer stemness and drug resistance via downregulation of the AREG-EGFR-ERK pathway. Oncogenesis..

[21] Lavon I, Zrihan D, Granit A, Einstein O, Fainstein N, Cohen MA, Cohen MA, Zelikovitch B, Shoshan Y, Spektor S (2010). Gliomas display a microRNA expression profile reminiscent of neural precursor cells. Neuro Oncol..

[22] Lages E, Guttin A, El Atifi M, Ramus C, Ipas H, Dupre I, Rolland D, Salon C, Godfraind C, deFraipont F (2011). MicroRNA and target protein patterns reveal physiopathological features of glioma subtypes. PLoS ONE.

[23] Rao SA, Santosh V, Somasundaram K (2010). Genome-wide expression profiling identifies deregulated miRNAs in malignant astrocytoma. Mod Pathol.

[24] Que T, Song Y, Liu Z, Zheng S, Long H, Li Z, Liu Y, Wang G, Liu Y, Zhou J (2015). Decreased miRNA-637 is an unfavorable prognosis marker and promotes glioma cell growth, migration and invasion via direct targeting Akt1. Oncogene.

[25] Sun G, Cao Y, Shi L, Sun L, Wang Y, Chen C, Wan Z, Fu L, You Y (2013). Overexpressed miRNA-137 inhibits human glioma cells growth by targeting Rac1. Cancer Biother Radiopharm..

[26] Lian S, Shi R, Bai T, Liu Y, Miao W, Wang H, Liu X, Fan Y (2013). Anti-miRNA-23a oligonucleotide suppresses glioma cells growth by targeting apoptotic protease activating factor-1. Curr Pharm Des.

[27] Li Y, Xu J, Chen H, Bai J, Li S, Zhao Z, Shao T, Jiang T, Ren H, Kang C (2013). Comprehensive analysis of the functional microRNA-mRNA regulatory network identifies miRNAsignatures associated with glioma malignant progression. Nucleic Acids Res.

[28] Fan B, Jiao BH, Fan FS, Lu SK, Song J, Guo CY, Yang JK, Yang L (2015). Downregulation of miR-95-3p inhibits proliferation, and invasion promoting apoptosis of glioma cells by targeting CELF2. Int J Oncol..

[29] Guo W, Dong Z, Liu S, Qiao Y, Kuang G, Guo Y, Shen S, Liang J (2017). Promoter hypermethylation-mediated downregulation of miR-770 and its host gene MEG3, a long non-coding RNA, in the development of gastric cardia adenocarcinoma. Mol Carcinog.

[30] Zhao H, Yu X, Ding Y, Zhao J, Wang G, Wu X, Jiang J, Peng C, Guo GZ, Cui S (2016). MiR-770-5p inhibits cisplatin chemoresistance in human ovarian cancer by targeting ERCC2. Oncotarget..

[31] Malumbres M, Harlow E, Hunt T, Hunter T, Lahti JM, Manning G, Morgan DO, Tsai LH, Wolgemuth DJ (2009). Cyclin-dependent kinases: a family portrait. Nat Cell Biol.

[32] Malumbres M, Barbacid M (2009). Cell cycle, CDKs and cancer: a changing paradigm. Nat Rev Cancer.

[33] Gu W, Wang C, Li W, Hsu FN, Tian L, Zhou J, Yuan C, Xie XJ, Jiang T, Addya S (2013). Tumor-suppressive effects of CDK8 in endometrial cancer cells. Cell Cycle.

[34] Malumbres M, Barbacid M (2005). Mammalian cyclindependent kinases. Trends Biochem Sci.

[35] Alarcón C, Zaromytidou AI, Xi Q, Gao S, Yu J, Fujisawa S, Barlas A, Miller AN, Manova-Todorova K, Macias MJ (2009). Nuclear CDKs drive Smad transcriptional activation and turnover in BMP and TGF-beta pathways. Cell.

[36] Xu W, Wang Z, Zhang W, Qian K, Li H, Kong D, Li Y, Tang Y (2015). Mutated K-ras activates CDK8 to stimulate the epithelial-to-mesenchymal transition in pancreatic cancer in part via the Wnt/β-catenin signaling pathway. Cancer Lett..

[37] Brägelmann J, Klümper N, Offermann A, von Mässenhausen A, Böhm D, Deng M, Queisser A, Sanders C, Syring I, Merseburger AS (2017). Pan-cancer analysis of the mediator complex transcriptome identifies CDK19 and CDK8 as therapeutic targets in advanced prostate cancer. Clin Cancer Res.

[38] Li XY, Luo QF, Wei CK, Li DF, Li J, Fang L (2014). MiRNA-107 inhibits proliferation and migration by targeting CDK8 in breast cancer. Int J Clin Exp Med..

[39] Firestein R, Bass AJ, Kim SY, Dunn IF, Silver SJ, Guney I, Freed E, Ligon AH, Vena N, Ogino S (2008). CDK8 is a colorectal cancer oncogene that regulates beta-catenin activity. Nature.

[40] Kim MY, Han SI, Lim SC (2011). Roles of cyclin-dependent kinase 8 and β-catenin in the oncogenesis and progression of gastric adenocarcinoma. Int J Oncol.

[41] Morris EJ, Ji JY, Yang F, Di Stefano L, Herr A, Moon NS, Kwon EJ, Haigis KM, Näär AM, Dyson NJ (2008). E2F1 represses beta-catenin transcription and is antagonized by both pRB and CDK8. Nature.

[42] Kim S, Xu X, Hecht A, Boyer TG (2006). Mediator is a transducer of Wnt/beta-catenin signaling. J Biol Chem.

[43] Carrera I, Janody F, Leeds N, Duveau F, Treisman JE (2008). Pygopus activates Wingless target gene transcription through the mediator complex subunits Med12 and Med13. Proc Natl Acad Sci USA.

[44] Zhao J, Ramos R, Demma M (2013). CDK8 regulates E2F1 transcriptional activity through S375 phosphorylation. Oncogene.

[45] Ohtsuka M, Ling H, Ivan C, Pichler M, Matsushita D, Goblirsch M, Stiegelbauer V, Shigeyasu K, Zhang X, Chen M (2016). H19 noncoding RNA, an independent prognostic factor, regulates essential Rb-E2F and CDK8-β-Catenin signaling in colorectal cancer. EBioMedicine..

[46] Zhao L, Liu Y, Tong D, Qin Y, Yang J, Xue M, Du N, Liu L, Guo B, Hou N (2017). MeCP2 promotes gastric cancer progression through regulating FOXF1/Wnt5a/β-Catenin and MYOD1/Caspase-3 signaling pathways. EBioMedicine..

[47] Lefèvre L, Omeiri H, Drougat L, Hantel C, Giraud M, Val P, Rodriguez S, Perlemoine K, Blugeon C, Beuschlein F (2015). Combined transcriptome studies identify AFF3 as a mediator of the oncogenic effects of β-catenin in adrenocortical carcinoma. Oncogenesis.

[48] Mohammed MK, Shao C, Wang J, Wei Q, Wang X, Collier Z, Tang S, Liu H, Zhang F, Huang J (2016). Wnt/β-catenin signaling plays an ever-expanding role in stem cell self-renewal, tumori-genesis and cancer chemoresistance. Genes Dis..

[49] Sinnberg T, Makino E, Krueger MA, Velic A, Macek B, Rothbauer U, Groll N, Pötz O, Czemmel S, Niessner H (2016). A nexus consisting of beta-Catenin and Stat3 attenuates braf inhibitor efficacy and mediates acquired resistance to vemurafenib. EBioMedicine..

[50] Yoon JH, Eun JW, Choi WS, Kim O, Nam SW, Lee JY, Park WS (2016). NKX6.3 is a transcription factor for Wnt/β-catenin and Rho-GTPase signaling-related genes to suppress gastric cancer progression. EBioMedicine..

[51] Chen Q, Yang D, Zong H, Zhu L, Wang L, Wang X, Zhu X, Song X, Wang J (2017). Growth-induced stress enhances epithelial-mesenchymal transition induced by IL-6 in clear cell renal cell carcinoma via the Akt/GSK-3β/β-catenin signaling pathway. Oncogenesis..

[52] Maddika S, Ande SR, Wiechec E, Hansen LL, Wesselborg S, Los M (2008). Akt-mediated phosphorylation of CDK2 regulates its dual role in cell cycle progression and apoptosis. J Cell Sci.

[53] Zhao LY, Zhang J, Guo B, Yang J, Han J, Zhao XG, Wang XF, Liu LY, Li ZF, Song TS (2013). MECP2 promotes cell proliferation by activation ERK1/2 and inhibiting p38 activity in human hepatocellular carcinoma HEPG2 cells. Cell Mol Biol (Noisy-le-grand).

